# Accuracy of Enzyme-Linked Immunosorbent Assays (ELISAs) in Detecting Antibodies against *Mycobacterium leprae* in Leprosy Patients: A Systematic Review and Meta-Analysis

**DOI:** 10.1155/2018/9828023

**Published:** 2018-11-25

**Authors:** Omar Ariel Espinosa, Silvana Margarida Benevides Ferreira, Fabiana Gulin Longhi Palacio, Denise da Costa Boamorte Cortela, Eliane Ignotti

**Affiliations:** ^1^Post Graduation Program in Health Science, Faculty of Medicine, Federal University of Mato Grosso (UFMT), Cuiaba, Mato Grosso, Brazil; ^2^Department of Medicine, Faculty of Health Sciences, State University of Mato Grosso (UNEMAT), Caceres, Mato Grosso, Brazil; ^3^Cuiabá University (UNIC), Cuiaba, Mato Grosso, Brazil; ^4^Post Graduation Program in Nursing, Faculty of Nursing, Federal University of Mato Grosso (UFMT), Cuiaba, Mato Grosso, Brazil; ^5^The Brazilian Centre for Evidence-based Healthcare: A Joanna Briggs Institute Centre of Excellence, Sao Paulo, Brazil; ^6^Department of Nursing, Faculty of Health Sciences, State University of Mato Grosso (UNEMAT), Caceres, Mato Grosso, Brazil

## Abstract

IgM against *Mycobacterium leprae* may be detected by enzyme-linked immunosorbent assays (ELISAs) based on phenolic glycolipid I (PGL-I) or natural disaccharide octyl bovine serum albumin (ND-O-BSA) as antigens, and the IgG response can be detected by an ELISA based on lipid droplet protein 1 (LID-1). The titers of antibodies against these antigens vary with operational classification. The aim of this study was to compare the accuracy of ELISAs involving PGL-I and ND-O-BSA with that involving LID-1. We included studies that analyze multibacillary and paucibacillary leprosy cases and evaluate the diagnostic accuracy of ELISAs based on LID-1 and/or PGL-I or ND-O-BSA as antigens to measure antibody titers against *M. leprae*. Studies were found via PubMed, the Virtual Health Library Regional Portal, Literatura Latino-Americana e do Caribe em Ciências da Saúde, Índice Bibliográfico Espanhol de Ciências de Saúde, the Brazilian Society of Dermatology, National Institute for Health and Clinical Excellence, Cochrane Library, Embase (the Elsevier database), and Cumulative Index to Nursing and Allied Health Literature. The Quality Assessment of Diagnostic Accuracy Studies served as a methodological validity tool. Quantitative data were extracted using the Standards for Reporting of Diagnostic Accuracy. Sensitivity, specificity, and a diagnostic odds ratio were calculated, and a hierarchical summary receiver-operating characteristic curve and forest plots were constructed. The protocol register code for this meta-analysis is PROSPERO 2017: CRD42017055983. Nineteen studies were included. ND-O-BSA showed better overall performance in terms of sensitivity, specificity, positive and negative likelihood ratios, and diagnostic odds ratio when compared with PGL-I and LID-1. The multibacillary group showed better performance on these parameters (than the paucibacillary group did), at 94%, 99%, 129, 0.05, and 2293, respectively. LID-1 did not provide any advantage regarding the overall estimate of sensitivity in comparison with PGL-I or ND-O-BSA.

## 1. Introduction

Leprosy is a chronic infectious disease caused by *Mycobacterium leprae* (a microorganism that mainly affects the skin and peripheral nerves) and is considered one of the six most dangerous diseases in developing countries by the World Health Organization (WHO) [1]. Leprosy diagnosis is based on the presence of at least one of the following three cardinal signs: definite loss of sensation in a pale or reddish skin patch, a thickened or enlarged peripheral nerve with loss of sensation and/or weakness of the muscles innervated by that nerve, and the presence of acid-fast bacilli in a slit-skin smear [2].

A continual and slow reduction in the number of new cases has been observed during the last decade, even though more than 200,000 new cases are diagnosed every year. India, Brazil, and Indonesia represent 80% of all cases [3].

A case of leprosy is defined as an individual that has a skin lesion consistent with leprosy, with definite sensory loss, with or without thickened nerves, and/or with positive skin smears [4]. Cases of leprosy are classified operationally as either paucibacillary leprosy (PB) or multibacillary leprosy (MB) depending on the number of skin lesions [5]. Early diagnosis and appropriate treatment of leprosy patients are essential conditions for stopping the transmission and reducing the physical and social consequences of the disease [4].

The discovery of phenolic glycolipid I (PGL-I) in 1980, a specific component of *M. leprae*, and its use in serological assays in patients with leprosy in 1981 are major advances in serological research on the disease [6–8]. Due to the glycolipid nature of PGL-I, the humoral immune response of leprosy patients predominantly involves IgM [9]. The detection of these IgM antibodies represents the best-evaluated and standardized serological test for leprosy [10–15]. In addition to native PGL-I, IgM levels can be measured by means of a synthetic mimotope: a natural disaccharide linked to bovine serum albumin (ND-O-BSA) [15–17]. The IgG response to *M. leprae* can be measured using LID-1 as an antigen: a chimeric protein generated by the fusion of antigens ML0405 and ML2331 [18]. Because it was reported early on that individuals with a high bacillary load have a high IgM titer against PGL-I [19], even in the chronic stage of the disease, the accuracy of the tests based on PGL-I (native or synthetic) and LID-1 has been compared previously in several studies, with the aim of identifying an adequate test for serological diagnosis [15–18, 20–24].

The titers of antibodies against PGL-I, ND-O-BSA, and LID-1 vary, with the clinical presentation being the strongest in MB patients and the weakest or absent in PB patients. The bacterial index may also correlate with antibody titers [20–22].

The Guidelines for the Diagnosis, Treatment and Prevention of Leprosy (WHO) warn that studies of the most commonly used ELISA and lateral flow tests show low sensitivity for PB leprosy, which is often harder to diagnose clinically than MB leprosy. Based on currently available evidence, newer ELISA and other laboratory tests do not represent a clear advantage over current standard diagnostic methods [25].

To date, a number of studies have used ELISAs based on PGL-I, ND-O-BSA, or LID-1 as antigens. The successful implementation of these methods reflects the good performance of these tests. Nonetheless, sensitivity and specificity of these assays vary depending on the geographic origin of the population studied [21]. Therefore, our aims were to conduct a meta-analysis of studies on the accuracy of the available serological tests and to summarize the accuracy of these tests in detecting antibodies against *M. leprae*. The aims were achieved successfully.

## 2. Methods

The protocol for this meta-analysis was published in the international prospective register of systematic reviews (PROSPERO 2017: CRD42017055983) before its implementation and is described in Supplementary Materials (Text S1). The protocol and final report were developed based on the Cochrane Handbook for Systematic Reviews of Diagnostic Test Accuracy [26].

### 2.1. The Review Question/Objective

What is the diagnostic accuracy of the commercially available ELISA based on antigen LID-1 as compared to ELISAs based on native antigen PGL-I or synthetic antigen ND-O-BSA for the detection of antibodies against *M. leprae* in patients with leprosy?

More specifically, we performed a meta-analysis of studies on the diagnostic test accuracy of PGL-I, ND-O-BSA, and LID-1 ELISAs to obtain summary points for the accuracy values of the assays for antibodies against *M. leprae*.

### 2.2. Inclusion Criteria

The mnemonic PIRD (*p*articipants, *i*ndex test, *r*eference test, and *d*iagnosis of interest) was employed for the inclusion criteria as recommended for systematic reviews of diagnostic test accuracy [26]. Studies were included that dealt with MB and PB leprosy cases and evaluated the diagnostic accuracy of ELISAs based on LID-1 and/or PGL-I or ND-O-BSA antigens to measure antibody titers against *M. leprae*.

The gold standard for the diagnosis of leprosy is based on clinical diagnosis. Therefore, only studies that selected and classified patients with leprosy on the basis of clinical diagnosis were included.

### 2.3. Types of Included Studies

The studies had to have any epidemiological design that afforded a detailed measure of sensitivity, specificity, and receiver-operating characteristic (ROC) curves.

### 2.4. The Search Strategy

This study was guided by the Preferred Reporting Items for Systematic Reviews and Meta-Analyses (PRISMA) protocol standard proposed by the Cochrane Collaboration® [27]. A three-step search strategy was utilized in this review. First, an initial limited search of Medline was performed by searching for MeSH index terms and related keywords. This search involved an analysis of words contained in the title and abstract and index terms used to describe the article. Second, another search involving all the identified keywords and index terms was performed across all the included databases. Third, a reference list of all dissertations with clearly detailed accurate values was considered. Studies published since 1982—the year when the first ELISA based on the PGL-I antigen was developed to detect antibodies against *M. leprae*—until February 2018 were considered for inclusion in this review. Moreover, only published studies were included because these studies were evaluated by external reviewers. The search strategy can be found in Supplementary Materials (Text S1).

Database searching was carried out in PubMed, which includes Medline and other health databases; in the Virtual Health Library Regional Portal (VHL Regional Portal), which includes Medline, Literatura Latino-Americana e do Caribe em Ciências da Saúde (LILACS), Índice Bibliográfico Espanhol de Ciências de Saúde (IBECS), and other health databases; via the Brazilian Society of Dermatology; at the National Institute for Health and Clinical Excellence (NICE); in the Cochrane Library; in Embase, the Elsevier database; and in the Cumulative Index to Nursing and Allied Health Literature (CINAHL). The databases used to search dissertations as a source of gray literature were Google Scholar and EVIPNet (WHO). The MeSH terms were Leprosy, *Mycobacterium leprae,* Serology, Enzyme-Linked Immunosorbent Assay, Leprosy Multibacillary, and Leprosy Paucibacillary. The keywords were LID-1, PGL-I, ND-O, NDO, IDR1, Specificity, Sensitivity, and Measurement Accuracy. The terms were combined via the boolean operators “AND” and/or “OR” to compose the search strings.

### 2.5. Assessment of Methodological Quality

The documents selected for retrieval were assessed by two independent reviewers for methodological validity prior to inclusion in this study, by means of standardized critical appraisal instruments from the Quality Assessment of Diagnostic Accuracy Studies (QUADAS 2), which was released in 2011 after revision of the original QUADAS. The QUADAS tool consists of four key domains that evaluate patient selection, an index test, reference standard, and flow and timing (flow of patients through the study and timing of the index tests and reference standard). Each domain is assessed in terms of the risk of bias, and the first three domains are also evaluated in terms of concerns about applicability [28, 29]. Any disagreements between the reviewers were resolved either through discussion or based on the opinion of a third reviewer.

### 2.6. Data Extraction

Quantitative data were extracted from papers according to the Standards for Reporting of Diagnostic Accuracy (STARD) [30, 31]. A 2 × 2 table was compiled to classify the data as true positive, false positive, true negative, and false negative.

### 2.7. Data Synthesis

STATA SM/64 (Version 13.1; College Station, TX) with MIDAS and METANDI commands was used for the meta-analysis.

Sensitivity, specificity, positive and negative likelihood ratios (LR+ and LR−), and the diagnostic odds ratio (DOR), with a confidence interval (CI) of 95%, were calculated for each study, and subsequently, the results were combined.

Two forest plots were generated side by side: one for sensitivity and the other for specificity showing the means and 95% CIs of each selected primary study. Through summary receiver-operating characteristic (SROC) curves, the presence or absence of heterogeneity was identified. The meta-analysis was performed based on the hierarchical model of summary receiver-operating characteristic (HSROC) curves [32]. The HSROC curve provides information on the overall performance (sensitivity, specificity, LR+, LR−, and DOR) of a test via different thresholds.

To evaluate the potential of publication bias, Deeks' funnel plot was constructed, with *p* < 0.05 indicating the presence of publication bias [33]. Fagan's nomogram, conceived to provide posttest probability, was employed to estimate clinical utility of the test values and is based on LR+ and LR− obtained from the meta-analysis [34].

## 3. Results

**Figure 1:**
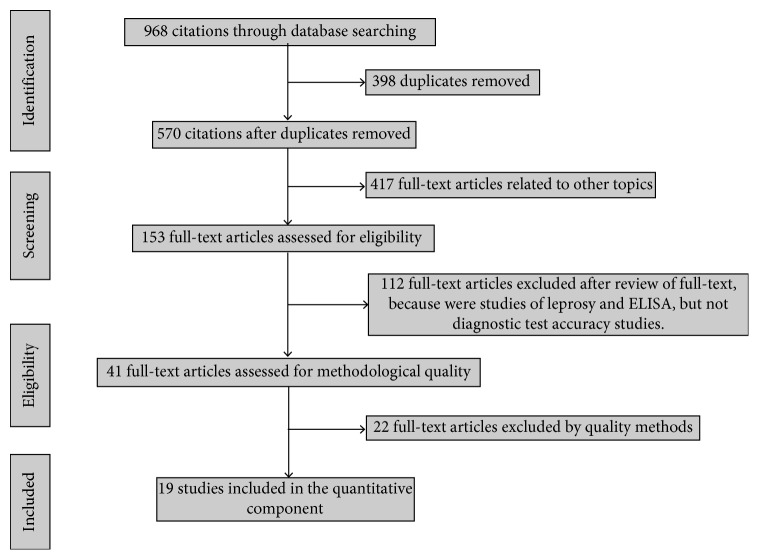
A flowchart of the steps performed in the systematic review and meta-analysis.

Our search yielded 968 citations related to leprosy through the combined application of descriptors in the databases described above. After the eligibility criteria (duplicate texts, articles related to other topics, and text excluded for review criteria or quality methods), 19 baseline studies remained. These studies evaluated the diagnostic accuracy of antigens PGL-I, ND-O-BSA, or LID-1 ELISAs and were included in this meta-analysis after critical appraisal of methodological quality [16–18, 22, 24, 35–48]. The results of our search strategy are shown in a PRISMA flowchart (Figure 1). Excluded studies are summarized in Table S1.

**Figure 2:**
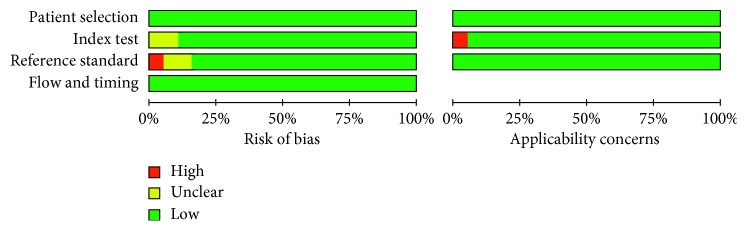
Assessment of methodological quality domains in all the studies. Proportions of studies rated as “high,” “unclear,” and “low” are presented.

The evaluation of methodological quality revealed that the studies included in this meta-analysis had a “low risk of bias” in patient selection and flow and time domains. Some of the selected studies were “at risk of bias” in the index test (10.5%) and the reference standard domains (15.7%). On the contrary, patient selection and the reference standard showed a “low applicability concern.” Only 5.2% of the selected studies yielded “applicability concerns” in the index test (Figure 2). The methodological quality summary bias risk concern and applicability of each domain for each included study are presented in Figure S1. The data extracted from the final selection are given in Table S2.

In the 19 studies included in this meta-analysis, 5512 ELISAs were carried out. These assays were performed on patients classified as MB (33.3%), PB (22.2%), and epidemiological control (44.5%). Concerning the geographic distribution, the samples were collected in Brazil (59%), China (14%), Philippines (11.2%), French Polynesia (8.8%), Spain (2%), Thailand (3%), and Nepal and Australia (2%). The distribution of performed ELISAs by antigen was as follows: PGL-I, 31.5%; ND-O-BSA, 42.1%; and LID-1, 26.4%. The data extracted from the selected studies are given in Table 1.

### 3.1. Effects of Clinical Manifestations of Leprosy on the Accuracy of Tests

To verify whether leprosy patient groups varied significantly in the performance of the *M. leprae* antigen ELISAs (PGL-I, ND-O-BSA, and LID-1), we carried out a global estimate of the accuracy of each test by group (MB and PB).

**Figure 3:**
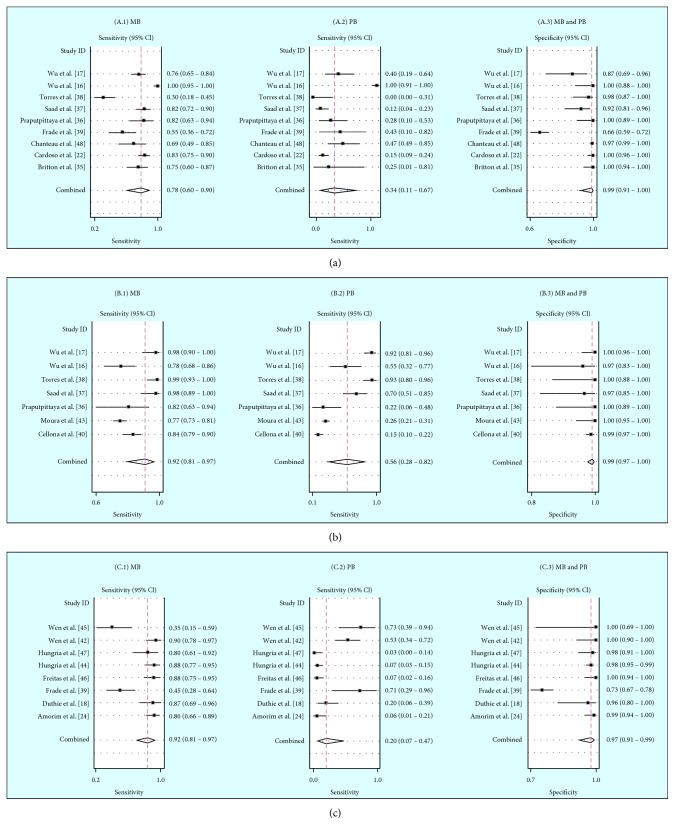
A forest plot of sensitivity of ELISAs by antigen: (a) PGL-I, (b) ND-O-BSA, and (c) LID-1, according to each studied group (MB (A.1, B.1, and C.1) and PB (A.2, B.2, and C.2)). The same specificity was found in both groups for the three ELISAs (A.3, B.3, and C.3). The circle in a square represents sensitivity and specificity, and the horizontal line represents the point estimate (95% CI for each study). Diamonds represent the combined value estimate (95% CI).

A forest plot of sensitivity and specificity of PGL-I in the MB group revealed sensitivity values ranging from 30% to 100% and specificity values from 66% to 100%. The combined sensitivity and specificity were 78% (95% CI 60–90) and 99% (95% CI 91–100), respectively. The sensitivity values for the PB group ranged from 12% to 100%, and the combined sensitivity was 34% (95% CI 11–67; Figure 3(a)).

In relation to the ND-O-BSA antigen, the MB group sensitivity values ranged from 77% to 100%, and specificity values ranged from 97% to 100%. The combined sensitivity and specificity were 92% (95% CI 81–97) and 99% (95% CI 98–100), respectively. In the PB group, sensitivity values ranged from 15% to 93%, and the combined sensitivity was 56% (95% CI 28–82; Figure 3(b)).

Finally, in terms of the LID-1 antigen, the MB group showed sensitivity values ranging from 35% to 90% and specificity values from 73% to 100%. The combined sensitivity and specificity were 80% (95% CI 66–89) and 97% (95% CI 93–100), respectively. The PB group showed sensitivity values ranging from 3% to 73%, and the combined sensitivity was 20% (95% CI 7–47; Figure 3(c)).

For all ELISA antigens, the combined specificity in the MB group was the same as that in the PB group.

### 3.2. Publication Bias and Heterogeneity

Deeks' funnel plot was constructed to analyze the potential publication bias for each antigen-specific ELISA in both patient groups. PGL-I Deeks' funnel plots did not reveal any publication bias in the two groups (*p*=0.63 and 0.69 for groups MB and PB, respectively). For the ND-O-BSA antigen, only Deeks' funnel plot in the MB group did not show publication bias (*p*=580). On the contrary, studies on the LID-1 antigen showed a publication bias risk in both the MB and PB groups (Figure S2).

The SROC curves for each ELISA antigen revealed a range of 87–100% for the area under the curve (AUC), with a 95% confidence contour and 95% prediction contour for each population studied (Figure S3). The SROC curves did not show heterogeneity among the included studies.

### 3.3. Accuracy of ELISAs in Detecting *M. leprae*

By means of the HSROC curves, the accuracy of each type of *M. leprae* ELISA based on different antigens was evaluated, and a summary point was generated for each population under study (Table 2). When we evaluated the accuracy of PGL-I ELISA assays, acceptable performance was observed only in the MB group. The respective sensitivity, LR+, LR−, and DOR values were as follows: MB group 78% (95% CI 60–90), 90 (95% CI 8–1023), 0.22 (95% CI 0.11–0.44), and 408 (95% CI 23–7041) and PB group 34% (95% CI 11–67), 16 (95% CI 2–121), 0.67 (95% CI 0.42–1), and 22 (95% CI 2.4–247; Figure S4A). ELISAs based on ND-O-BSA showed better performance in both groups: 94% (95% CI 78–98), 129 (95% CI 42–390), 0.05 (95% CI 0.01–0.23), and 2293 (95% CI 279–18844) in group MB and 56% (95% CI 27–81), 76 (95% CI 21–274), 0.43 (95% CI 0.21–0.87), and 174 (95% CI 39–1013) in group PB, respectively (Figure S4B). LID-1 ELISAs showed the worst performance among the three antigens. The respective sensitivity, LR+, LR-, and DOR accuracy data were as follows: in the MB group, 79% (95% CI 66–88), 26 (95% CI 8–90), 0.2 (95% CI 0.11–0.37), and 127 (95% CI 22–721), and in the PB group, 20% (95% CI 7–46), 8.0 (95% CI 3–24), 0.81 (95% CI 0.64–1), and 9.8 (95% CI 2.8–33; Figure S4C). Specificity values were between 97% and 99% in each type of ELISA and in each group analyzed.

### 3.4. DOR and Posttest Probability

DORs were considerably higher in the MB group than in the PB group for all the antigens used in the ELISAs.

**Figure 4:**
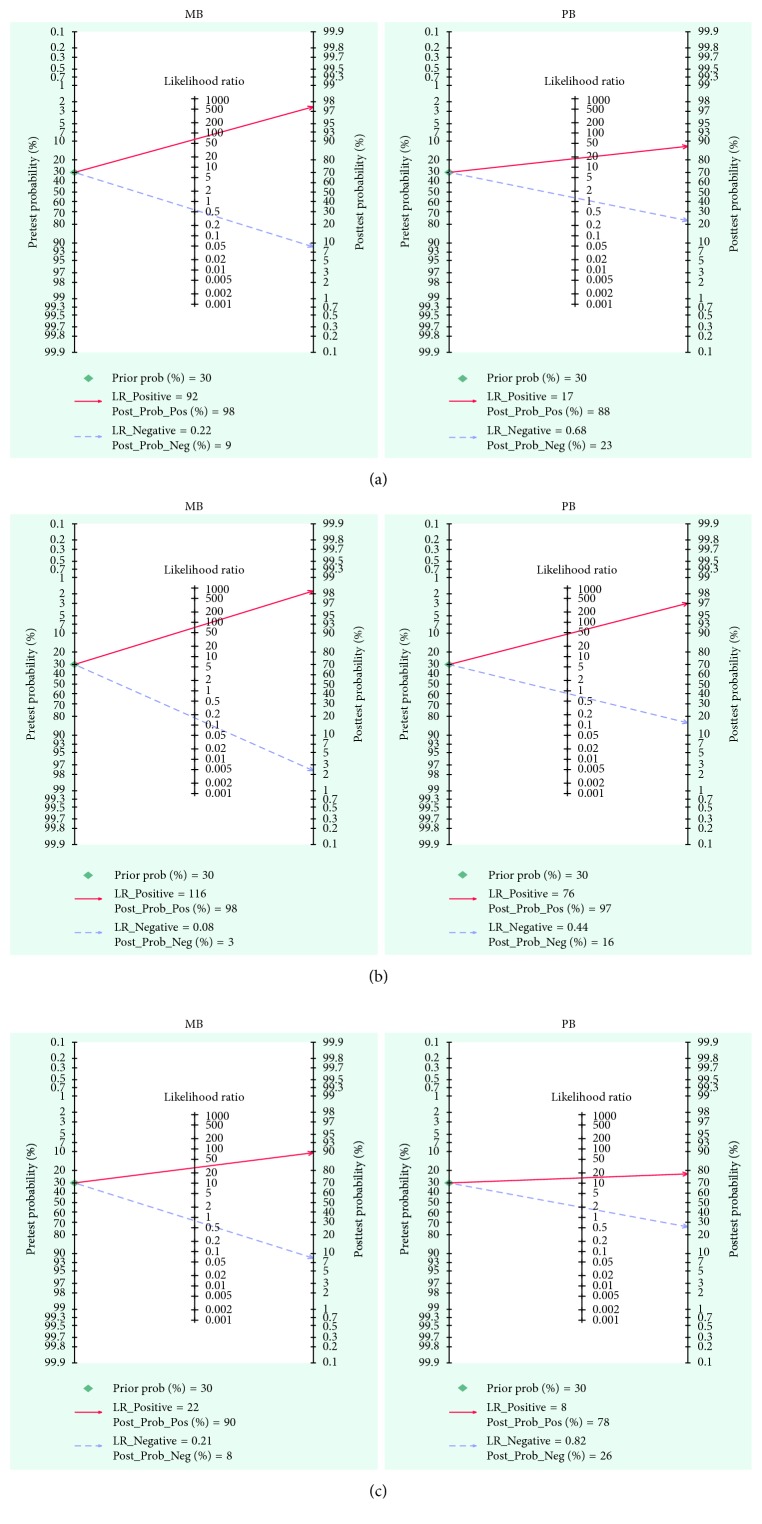
Fagan's nomogram and the posttest probabilities. Fagan's nomogram was built with a prevalence of 30% for household contacts of leprosy patients in an endemic area. If a patient tests positive, the posttest probability that they truly have leprosy would be (a) PGL-I ELISA: 98% for group MB and 88% for group PB; (b) ND-O-BSA ELISA: 98% for group MB and 97% for group PB; and (c) LID-1 ELISA: 90% for group MB and 78% for group PB (solid red line). On the contrary, if this patient tests negative, the posttest probability of having the disease and not being detected would be (a) PGL-I ELISA: 9% for group MB and 23% for group PB; (b) ND-O-BSA ELISA: 3% for group MB and 16% for group PB; and (c) LID-1 ELISA: 8% for group MB and 26% for group PB (dotted blue line).

Fagan's nomogram was built to obtain posttest probability, for which we performed a simulation with a prevalence of 30% for household contacts of leprosy patients from endemic areas in accordance with the included studies. Thus, the probability of someone having the disease and not being detected by the PGL-I ELISA was 9% and 23% in the MB and PB groups, respectively. In contrast, the posttest probability of sick patients with a positive test was 98% and 88% for groups MB and PB (Figure 4(a)). Similarly, the probability of someone having the disease and not being detected by the NO-O-BSA ELISA was 3% and 16% in groups MB and PB, respectively, whereas these values were 98% and 97%, respectively, for patients with leprosy (Figure 4(b)). For the LID-1 ELISA, the posttest probabilities were 8% and 26% for an individual having the disease and not being diagnosed and 90% and 78% for leprosy patients with a positive test in groups MB and PB, respectively (Figure 4(c)).

## 4. Discussion

Most of the ELISAs performed in the included studies in this systematic review and meta-analysis were performed in Brazil (59%) and Asia (28.2%, predominantly in China): countries that have different epidemiological profiles [4]. These studies revealed variations in sensitivity and specificity depending on the ELISA antigen and the patient group (MB or PB). These variations may be related to the strains found in each region and immune responses of the patients.

In the present meta-analysis, studies that analyzed ELISA tests involving the ND-O-BSA antigen indicated sensitivity (77–100% for group MB and 15–93% for group PB) and specificity (97–100%) ranges that are more favorable than did studies on PGL-I and LID-1 ELISAs. Sensitivity values among studies from different regions and among studies from the same regions showed great differences, for both the MB and PB groups, as reported previously [21]. Even studies that were designed by the same authors and conducted in the same regions produced different sensitivity values [16, 17]. Specificity values were more similar among the studies analyzed in both groups, MB and PB.

A general diagnostic test accuracy estimate was carried out for each ELISA antigen in both leprosy groups. The HSROC curves showed better sensitivity (94% (95% CI 78–98) for MB and 56% (95% CI 17–81) for PB) and specificity (99% (95% CI 97–100)) for the ND-O-BSA antigen. In our results, the ELISAs using PGL-I were not subject to conclusive publication bias in either of the groups studied (MB or PB). As for the ELISA involving the ND-O-BSA antigen, only the MB group showed publication bias.

Sensitivity and specificity found for each ELISA matched the accuracy reported by other authors [15, 43, 44, 49]. On the contrary, when we compared the performance reported in these studies with our results, there was no consensus regarding a superior antigen for leprosy ELISAs. This finding may be due to the fact that most of the studies were conducted with conventional ELISAs made in-house and due to other factors like sampling time, sample transport, and sample preservation, which may cause test performance variations. Additionally, there is no standardized cutoff value for any of these ELISA antigens or for either group of patients with leprosy. Nevertheless, we can conclude that all the analyzed antigens have better diagnostic accuracy for MB leprosy, as reported elsewhere in the literature [15, 21, 23, 24, 43, 45, 49, 50]. Very divergent accuracy results in the group of PB patients were found. Based on the estimated median sensitivities found in this patient group, negative tests are not that useful for ruling out PB leprosy patients. Using these serologic tests, PB patients can be diagnosed as negative when they really are not.

The absence of added value for the use of LID-1 was also observed in a recent study, where the researchers detected antibodies against the PGL-I antigen in patients with leprosy by rapid tests [51].

Owing to the presence of anti-BSA antibodies, which may interfere with the test results [52], the ND-O antigen conjugated to human serum albumin (HSA) has been used in ELISAs [15, 43, 49]. Nevertheless, these studies suggest that the BSA or HSA carrier protein of the antigen does not significantly influence the anti-PGL-I seropositivity of the groups under study [15, 43]. The primary literature on ELISAs using the ND-O-HSA antigen was not included because only three studies were found, and at least four are required for a reliable meta-analysis.

The DORs obtained here were higher on average in the MB group than in the PB group. The DOR varies from zero to infinity, with higher values denoting a better discriminatory diagnostic test. Additionally, posttest probability (Fagan's nomogram) was high, specifically within the MB patient group; for each antigen included in our analysis, this result is indicative of good clinical utility of an ELISA as a supplement to clinical diagnosis.

There are some limitations of our study. First of all, there is no standard cutoff value for any ELISA antigen analyzed here and for either group of patients under study (MB or PB). Although this situation did not hamper implementation of our meta-analysis, accuracy variations can occur due to differences in the number of true or false positives or negatives among the studies. Management of outliers varies among authors and affects the measurement of accuracy parameters, resulting in a wide range of sensitivity and specificity estimates across studies, as shown in our study. Second, although the specificity was almost 100%, only a few authors included groups of controls of patients with other diseases such as tuberculosis, and most authors used endemic control samples and a few samples of nonendemic controls. Nevertheless, there was a risk of publication bias in the studies on the ND-O-BSA antigen in the PB group. Few studies indicated whether the samples were from primary or secondary infections, or whether the patients received treatment. Finally, due to different amounts of patient information in the included studies, the data were not divided into additional groups based on other variables, e.g., gender or age.

## 5. Conclusion

In this meta-analysis, in the MB group, the LID-1 ELISA did not show any advantage with respect to the overall sensitivity estimate (79% (95% CI 66–89)) when compared to native antigen PGL-I (78% (95% CI 60–90)) or to synthetic antigen ND-O-BSA (94% (95% CI 81–97)). Specificity of all the ELISAs in this group was close to 100% for all antigens, whereas in the PB group, all the assays showed lower sensitivity values as compared with the MP group in terms of detection of antibodies against *M. leprae*.

Our results confirm that traditional ELISAs have good accuracy in detecting MB leprosy and poor accuracy in detecting PB leprosy. The WHO research priorities for leprosy include new tools for early detection. To achieve this goal, it is important to have a standardized serological, molecular, or immunological assay that is applicable to different geographic regions with different epidemiological profiles and pathogen strains. In the future, these laboratory tools are expected to become important for the diagnosis of leprosy (MB and PB), for surveillance of household contacts, and for establishing health policy interventions.

## Figures and Tables

**Table 1:** A summary of the included studies.

Antigen	Journal	Year	Author	Country	Method	Dilution	Cut-off	TP/total		FN/total
							OD	MB	PB	EC
*PGL-1*										
	Aust. NZ J Med	1987	Britton	Australia and Nepal	Conventional	1/100	>0.15	33/44	1/4	0/60
	Int. J. Lepr.	1988	Wu	China	Conventional	1/200	>0.04	76/76	40/40	0/30
	Int. J. Lepr.	1990	Wu	China	Conventional	1/201	>0.2	70/90	11/20	1/30
	Asian Pac J Allergy Immunol.	1990	Praputpittaya	Thailand	Conventional	1/300	>0.056	23/28	5/18	0/33
	Mem. Inst. Oswaldo Cruz	1990	Saad	Brazil	Conventional	1/4000	>0.27	61/74	6/52	4/52
	Lepr. Rev.	1991	Chanteau	French Polynesia	Conventional	1/200	>0.2	20/21	8/23	9/414
	BMC Infect. Dis.	2013	Vaz Cardoso	Brazil	Conventional	1/300	>0.250	90/108	16/104	1/30
	Lepr. Rev.	2003	Torres	Spain	Conventional	1/300	>0.160	15/50	0/10	1/40
	PLoS Neglected Trop. Dis	2017	Frade	Brazil	Conventional	1/400	0.1	15/33	5/7	67/245
*ND-O-BSA*										
	Int. J. Lepr.	1988	Wu	China	Conventional	1/200	>0.05	76/76	37/40	0/30
	Int. J. Lepr.	1990	Wu	China	Conventional	1/201	>0.2	70/90	11/20	1/30
	Asian Pac. J. Allergy Immunol.	1990	Praputpittaya	Thailand	Conventional	1/300	>0.056	23/28	5/18	0/33
	Int. J. Lepr.	1993	Cellona	Philippines	Conventional	—	0.16	163/193	22/147	7/401
	Int. J. Lepr.	2002	Wu	China	Conventional	1/200	—	53/53	46/50	0/100
	Am. J. Trop. Med. Hyg.	2013	Wen	China	Conventional	—	>0.2	48/49	21/30	1/35
	J. Immunol. Methods	2014	Moura	Brazil	Conventional		>0.2	375/486	88/342	0/69
*+LID-1*										
	Clin. Vaccine Immunol.	2007	Duthie	Brazil	Conventional	1/1000	0.1	26/30	6/30	1/26
	Mem. Inst. Oswaldo Cruz	2012	Hungria	Brazil	Conventional	1/200	0.3	51/58	6/93	7/282
	Am. J. Trop. Med. Hyg.^*∗*^	2013	Wen	China	Conventional	—	>0.2	44/49	16/30	0/35
	Biomed Res. Int.	2014	Wen	China	Conventional	1/200	>2 × SD OD EC	7/20	8/11	0/10
	PLoS Neglected Trop. Dis	2016	Amorim	Brazil	Conventional	1/200	>3 × SD OD EC	58/68	5/32	1/98
	Diagn Microbiol. Infect. Dis.	2016	Freitas	Brazil	Conventional	1/200	0.3	42/48	4/60	0/62
	Diagn Microbiol. Infect. Dis.	2017	Hungria	Brazil	Conventional	1/200	0.3	24/30	1/38	2/61
	PLoS Neglected Trop. Dis.	2017	Frade	Brazil	Conventional	1/400	0.1	15/33	5/7	67/245

^*∗*^Type of sample used: plasma. All other studies used serum. TP/total = true positive/total of cases; FN/total = false negative/total of endemic control; EC = endemic control; +LID-1: Developed by Infectious Disease Research Institute (IDRI).

**Table 2:** Accuracy of ELISAs for detection of leprosy using different *M. leprae* antigens. Summary points of the HSROC curve accuracy for each *M. leprae* antigen used in the ELISAs for each population studied.

ELISA antigen/Op. class^*∗*^	Sensitivity %	95% CI	Specificity %	95% CI	LR+	95% CI	LR−	95% CI	DOR	95% CI
*PGL-I*										
MB	78	60–90	99	91–99	90	8–1023	0.22	0.11–0.44	408	23–7041
PB	34	11–67	97	89–99	16	2–121	0.67	0.42–1	22	2.4–247
*ND-O-BSA*										
MB	94	78–98	99	97–99	129	42–390	0.05	0.01–0.23	2293	279–18844
PB	56	27–81	99	98–99	76	21–274	0.43	0.21–0.87	174	39–1013
*LID-1*										
MB	79	66–88	97	91–99	26	8–90	0.2	0.11–0.37	127	22–721
PB	20	7–46	97	92–99	8	3.0–24	0.81	0.64–1	9.8	2.8–33

^*∗*^Op. class = operational classification; 95% CI = 95% confidence interval; LR− = negative likelihood; LR+ = positive likelihood ratio; DOR = diagnostic odds ratio.

## Supplementary Materials

Supplementary Materials Text S1: the protocol registered in Prospero International (PROSPERO 2017: CRD42017055983). Text S2: the literature search strategy. Table S1: a summary of the excluded studies. Table S2: the PRISMA checklist. Figure S1: methodological quality summary of the included studies. Figure S2: analysis of publication bias. Deeks' funnel plot for leprosy ELISAs is based on different antigens. Deeks' funnel plot (asymmetry test for publication bias) did not detect potential publication bias for (A) PGL-I (MB *p*=0.63 and PB *p*=0.69). (B) Only ND-O-BSA in the MB group did not show publication bias (*p*=58). (C) LID-1 showed publication bias in both groups of patients. Figure S3: analysis of heterogeneity. Summary ROC curve plots of sensitivity and specificity for PGL-I (A), ND-O-BSA (B), and LID-1 (C). Each large X represents an individual study in the meta-analysis. The summary operating point is a single sensitivity/specificity point estimated by the results of the studies. AUC = area under the curve. Figure S4: meta-analysis based on the hierarchical method. An HSROC plot displaying diagnostic accuracy results of the included studies by antigen in ELISAs: (A) PGL-I, (B) ND-O-BSA, and (C) LID-1 for different leprosy patient groups (MB and PB). The circle diameter (study estimate) is proportional to the weight given to each study. Summary sensitivity and specificity estimates are marked with a red square.
