# Supplementary material for: Accuracy of Enzyme-Linked Immunosorbent Assays (ELISAs) in Detecting Antibodies against Mycobacterium leprae in Leprosy Patients: A Systematic Review and Meta-Analysis
Source: Can J Infect Dis Med Microbiol. 2018 Nov 25;2018:9828023. doi: 10.1155/2018/9828023 (PMC6286776; doi:10.1155/2018/9828023)

National Institute for Health Research

UNIVERSITY of York
Centre for Reviews and Dissemination

Systematic review

1. * Review title.

Give the working title of the review, for example the one used for obtaining funding. Ideally the title should state succinctly the interventions or exposures being reviewed and the associated health or social problems. Where appropriate, the title should use the PI(E)COS structure to contain information on the Participants, Intervention (or Exposure) and Comparison groups, the Outcomes to be measured and Study designs to be included.

Accuracy of enzyme-linked immunosorbent assays (ELISAs) in detecting antibodies against Mycobacterium leprae in leprosy patients: A systematic review and meta-analysis

30 words remaining

2. Original language title.

For reviews in languages other than English, this field should be used to enter the title in the language of the review. This will be displayed together with the English language title.

50 words remaining

3. * Anticipated or actual start date.

Give the date when the systematic review commenced, or is expected to commence.

15/02/2017

4. * Anticipated completion date.

Give the date by which the review is expected to be completed.

15/02/2018

5. * Stage of review at time of this submission.

Indicate the stage of progress of the review by ticking the relevant Started and Completed boxes. Additional information may be added in the free text box provided.
Please note: Reviews that have progressed beyond the point of completing data extraction at the time of initial registration are not eligible for inclusion in PROSPERO. Should evidence of incorrect status and/or completion date being supplied at the time of submission come to light, the content of the PROSPERO record will be removed leaving only the title and named contact details and a statement that inaccuracies in the stage of the review date had been identified.
This field should be updated when any amendments are made to a published record and on completion and publication of the review.

The review has not yet started: No

Review stage	Started	Completed
Preliminary searches	Yes	Yes
Piloting of the study selection process	Yes	Yes
Formal screening of search results against eligibility criteria	Yes	Yes
Data extraction	Yes	Yes
Risk of bias (quality) assessment	Yes	Yes
Data analysis	Yes	Yes

Provide any other relevant information about the stage of the review here (e.g. Funded proposal, protocol not yet finalised).

6. * Named contact.

The named contact acts as the guarantor for the accuracy of the information presented in the register record.
Dr Espinosa

Email salutation (e.g. "Dr Smith" or "Joanne") for correspondence:

7. * Named contact email.

Give the electronic mail address of the named contact.
oaetmpan@gmail.com

8. Named contact address

Give the full postal address for the named contact.
Rua dos Aviadores nº163, ap 7. Cáceres, Mato Grosso, Brasil

9. Named contact phone number.

Give the telephone number for the named contact, including international dialling code.
+5565999995940

10. * Organisational affiliation of the review.

Full title of the organisational affiliations for this review and website address if available. This field may be completed as 'None' if the review is not affiliated to any organisation.
Federal University of Mato Grosso

Organisation web address:

http://www.ufmt.br/ufmt/site/

11. Review team members and their organisational affiliations.

Give the title, first name, last name and the organisational affiliations of each member of the review team. Affiliation refers to groups or organisations to which review team members belong.

Dr Omar Espinosa. Federal University of Mato Grosso
Dr Silvana Benevides. Federal University of Mato Grosso
Dr Eliane Ignotti. Mato Grosso State University
Dr Fabiana Gulin Longhi Palacio. Librarian of The Brazilian Centre for Evidence-based Healthcare: A Joanna Briggs Institute Centre of Excellence

Dr Denise da Costa Boamorte Cortela. Department of Medicine, Faculty of Health Sciences, State of University of Mato Grosso

12. * Funding sources/sponsors.

Give details of the individuals, organizations, groups or other legal entities who take responsibility for initiating, managing, sponsoring and/or financing the review. Include any unique identification numbers assigned to the review by the individuals or bodies listed.

EDITAL PPSUS- / FAPEMAT Nº 002-2013
PROCESSO Nº. 250393/2013

13. * Conflicts of interest.

List any conditions that could lead to actual or perceived undue influence on judgements concerning the main topic investigated in the review.

None

14. Collaborators.

Give the name and affiliation of any individuals or organisations who are working on the review but who are not listed as review team members.

15. * Review question.

State the question(s) to be addressed by the review, clearly and precisely. Review questions may be specific or broad. It may be appropriate to break very broad questions down into a series of related more specific questions. Questions may be framed or refined using PI(E)COS where relevant.

What is the diagnostic accuracy of the commercially available NDO-LID antigen based ELISA assay compared to ELISA based on the PGL-1 reference antigen for the detection of antibodies against M. leprae in patients with leprosy?

215 words remaining

16. * Searches.

Give details of the sources to be searched, search dates (from and to), and any restrictions (e.g. language or publication period). The full search strategy is not required, but may be supplied as a link or attachment.

Three-step search strategy will be utilized in this review. An initial limited search of MEDLINE will be performed searching the MeSH index terms and related keywords. A second search using all identified keywords and index terms will be made across all included databases. Thirdly, we will perform a search to find grey literature.

247 words remaining

17. URL to search strategy.

Give a link to the search strategy or an example of a search strategy for a specific database if available (including the keywords that will be used in the search strategies).

Alternatively, upload your search strategy to CRD in pdf format. Please note that by doing so you are consenting to the file being made publicly accessible.

Yes I give permission for this file to be made publicly available

18. * Condition or domain being studied.

Give a short description of the disease, condition or healthcare domain being studied. This could include health and wellbeing outcomes.

In this project we intend to conduct a systematic review and meta-analysis of the serological anti PGL-1 and NDO-LID-1 antigen based ELISA assay. No meta-analysis studies have been performed to evaluate the diagnostic accuracy of serological ELISA assay based on the detection of PGL-I and NDO-LID antibodies. These tests are used as diagnosis tools, classification of patients, treatment evolution, risk of recurrence and in the selection of contacts with higher risk to develop the disease

125 words remaining

19. * Participants/population.

Give summary criteria for the participants or populations being studied by the review. The preferred format includes details of both inclusion and exclusion criteria.

This review will consider studies that include patients with leprosy and household contacts.

187 words remaining

20. * Intervention(s), exposure(s).

Give full and clear descriptions or definitions of the nature of the interventions or the exposures to be reviewed.

Index Test

This review will consider studies that evaluate the diagnostic accuracy of ELISA assays using NDO-LID antigens.

183 words remaining

21. * Comparator(s)/control.

Where relevant, give details of the alternatives against which the main subject/topic of the review will be compared (e.g. another intervention or a non-exposed control group). The preferred format includes details of both inclusion and exclusion criteria.

Reference test

This review will consider studies that include the diagnostic accuracy of ELISA assays using PGL-1 antigens.

183 words remaining

22. * Types of study to be included.

Give details of the types of study (study designs) eligible for inclusion in the review. If there are no restrictions on the types of study design eligible for inclusion, or certain study types are excluded, this should be stated. The preferred format includes details of both inclusion and exclusion criteria.

Cross-sectional and longitudinal analytical studies will be included

142 words remaining

23. Context.

Give summary details of the setting and other relevant characteristics which help define the inclusion or exclusion criteria.

Exclusion Criteria.

Studies without Cutoff Value.

244 words remaining

24. * Main outcome(s).

Give the pre-specified main (most important) outcomes of the review, including details of how the outcome is defined and measured and when these measurement are made, if these are part of the review inclusion criteria.

Diagnosis of interest

This review will consider studies that include measure of titters of antibodies against M. leprae.

183 words remaining

Timing and effect measures

200 words remaining

25. * Additional outcome(s).

List the pre-specified additional outcomes of the review, with a similar level of detail to that required for main outcomes. Where there are no additional outcomes please state ‘None’ or ‘Not applicable’ as appropriate to the review

None

299 words remaining

Timing and effect measures

300 words remaining

26. Data extraction (selection and coding).

Give the procedure for selecting studies for the review and extracting data, including the number of researchers involved and how discrepancies will be resolved. List the data to be extracted.
Quantitative data will be extracted from papers included in the review using The STARD (Standards for Reporting of Diagnostic Accuracy). Any disagreements that arise between the reviewers will be resolved through discussion, or with a third reviewer.
263 words remaining

27. * Risk of bias (quality) assessment.

State whether and how risk of bias will be assessed (including the number of researchers involved and how discrepancies will be resolved), how the quality of individual studies will be assessed, and whether and how this will influence the planned synthesis.
Papers selected for retrieval will be assessed by two independent reviewers for methodological validity prior to inclusion in the review using standardized critical appraisal instruments from the QUADAS 2.
171 words remaining

28. * Strategy for data synthesis.

Give the planned general approach to synthesis, e.g. whether aggregate or individual participant data will be used and whether a quantitative or narrative (descriptive) synthesis is planned. It is acceptable to state that a quantitative synthesis will be used if the included studies are sufficiently homogenous.
Two forest plots are presented side by side: one for sensitivity and the other for specificity. Moreover, the number of true positives, false positives, true negatives and false negatives are also reported, as well as, where appropriate, any covariates (for instance the type of diagnostic test used). Summary ROC (SROC) curves will also be presented.
Through the graphs of paired forests or the SROC curve the presence or absence of heterogeneity will be identified. If there are differences in the diagnostic threshold between the studies, the forest charts will not be used to analyze the heterogeneity, in which case it will be estimated through the SROC curve. For more objective assessments of heterogeneity will be performed statistical tests of chi-square (Cochrane Q) and I-squared.
The meta-analysis will be performed based on the Bivariate method to estimate a summary of the parameters of the primary studies: sensitivity and specificity (Reitsma et al., 2009). To treat the variability in cutoff values, a Hierarchal Sumary Receiver Operating Characteristic (HSROC).
134 words remaining

29. * Analysis of subgroups or subsets.

Give details of any plans for the separate presentation, exploration or analysis of different types of participants (e.g. by age, disease status, ethnicity, socioeconomic status, presence or absence or co-morbidities); different types of intervention (e.g. drug dose, presence or absence of particular components of intervention); different settings (e.g. country, acute or primary care sector, professional or family care); or different types of study (e.g. randomised or non-randomised).
This will be determined by the homogenety/heterogenity of data.
241 words remaining

30. * Type and method of review.

Select the type of review and the review method from the lists below. Select the health area(s) of interest for your review.

Type of review

Cost effectiveness
No

Diagnostic
No

Epidemiologic
No

Individual patient data (IPD) meta-analysis
No

Intervention
No

Meta-analysis
Yes

Methodology
No

Narrative synthesis
No

Network meta-analysis
No

Pre-clinical
No

Prevention
No

Prognostic
No

Prospective meta-analysis (PMA)
No

Review of reviews
No

Service delivery
No

Synthesis of qualitative studies
No

Systematic review
Yes

Other
No

Health area of the review

Alcohol/substance misuse/abuse
No

Blood and immune system
No

Cancer
No

Cardiovascular
No

Care of the elderly
No

Child health
No

Complementary therapies
No

Crime and justice
No

Dental
No

Digestive system
No

Ear, nose and throat
No

Education

No

Endocrine and metabolic disorders
No

Eye disorders
No

General interest
No

Genetics
No

Health inequalities/health equity
No

Infections and infestations
No

International development
No

Mental health and behavioural conditions
No

Musculoskeletal
No

Neurological
No

Nursing
No

Obstetrics and gynaecology
No

Oral health
No

Palliative care
No

Perioperative care
No

Physiotherapy
No

Pregnancy and childbirth
No

Public health (including social determinants of health)
No

Rehabilitation
No

Respiratory disorders
No

Service delivery
No

Skin disorders
No

Social care
No

Surgery
No

Tropical Medicine
No

Urological
No

Wounds, injuries and accidents
No

Violence and abuse
No

31. Language.

Select each language individually to add it to the list below, use the bin icon to remove any added in error.
English

There is an English language summary.

32. Country.

Select the country in which the review is being carried out from the drop down list. For multi-national collaborations select all the countries involved.

Brazil

33. Other registration details.

Give the name of any organisation where the systematic review title or protocol is registered (such as with The Campbell Collaboration, or The Joanna Briggs Institute) together with any unique identification number assigned. (N.B. Registration details for Cochrane protocols will be automatically entered). If extracted data will be stored and made available through a repository such as the Systematic Review Data Repository (SRDR), details and a link should be included here. If none, leave blank.
50 words remaining

34. Reference and/or URL for published protocol.

Give the citation and link for the published protocol, if there is one

Give the link to the published protocol.

Alternatively, upload your published protocol to CRD in pdf format. Please note that by doing so you are consenting to the file being made publicly accessible.

Yes I give permission for this file to be made publicly available

Please note that the information required in the PROSPERO registration form must be completed in full even if access to a protocol is given.

35. Dissemination plans.

Give brief details of plans for communicating essential messages from the review to the appropriate audiences.
Leprosy and Tropical Medicine Congress; international publication.

Do you intend to publish the review on completion?

Yes

36. Keywords.

Give words or phrases that best describe the review. Separate keywords with a semicolon or new line. Keywords will help users find the review in the Register (the words do not appear in the public record but are included in searches). Be as specific and precise as possible. Avoid acronyms and abbreviations unless these are in wide use.
Leprosy

PGL-1
NDO-LID-1
ELISA
Accuracy
Household
LID-1
Mycobacterium leprae
Serology

37. Details of any existing review of the same topic by the same authors.

Give details of earlier versions of the systematic review if an update of an existing review is being registered, including full bibliographic reference if possible.

50 words remaining

38. * Current review status.

Review status should be updated when the review is completed and when it is published.
Please provide anticipated publication date

Review_Completed_not_published

39. Any additional information.

Provide any other information the review team feel is relevant to the registration of the review.

40. Details of final report/publication(s).

This field should be left empty until details of the completed review are available.

Give the link to the published review.

Strategy Search

Pubmed strategy search

(Leprosy OR “Leprosy, Multibacillary” OR “Leprosy, Paucibacillary”) AND (“Enzyme-Linked Immunosorbent Assay” OR ELISA OR Serology OR “Sensitivity and Specificity”) AND (“PGL-1” OR PGL1 OR “ND-O” OR NDO OR “LID-1” OR LID1 OR IDRI)

Embase strategy search

('leprosy'/exp OR 'multibacillary leprosy'/exp OR 'paucibacillary leprosy'/exp) AND ('serology'/exp OR 'enzyme linked immunosorbent assay'/exp OR 'measurement accuracy'/exp OR 'lid-1':ab,ti OR 'idr1':ab,ti OR 'specificity'/exp OR 'sensitivity'/exp) AND ('serology'/exp OR 'enzyme linked immunosorbent assay'/exp OR 'measurement accuracy'/exp OR 'pgl-1':ab,ti OR 'nd-o':ab,ti OR 'specificity'/exp OR 'sensitivity'/exp OR 'ndo':ab,ti) AND ('mycobacterium leprae'/exp OR 'lid-1':ab,ti OR 'pgl-1':ab,ti)

S1 Table. A summary of the excluded studies.

						Cut-off			
Antigen	Year	Author	Country	Method	Dilution	OD	EI	Sample №	Reasons
ND-O-BSA									
	1987	Chanteau	Polinésia	Conventional	1/250	-	-	724	It is not a accuracy study
	1992	Douglas	Philippines	Conventional	1/500	0.16		398	It is not a accuracy study
	2013	Qiong-Hua	China	Conventional	1/1000	-	-	116	It is not a accuracy study
PGL-1									
	1988	Burgess	Malawi	Conventional	1/100	0.85		102	It is not a accuracy study
	1993	H Lal	India	Conventional	-	0.115		50	It is not a accuracy study
	1995	Agdamag	Philippines	Conventional	-	-	-	90	Total of False Negative unclear
	1999	Kampirapap	Kampirapap	Conventional	1/8 - 1/16	-	-	156	Endemic controls were not included
	1999	Roche	Nepal	Conventional	1/300	0.2		174	Endemic controls were not included
	2001	Cho	Philippines	Conventional	1/300	-	-	100	It is not a accuracy study
	2011	Bazan-Furini	Brazil	Conventional	1/100	0.028	-	403	Total of False Negative unclear
	2011	Lobato	Brazil	Conventional	1/300	-	1.1	345	It is not a accuracy study
	2014	Duthie	Colombia & Philippines	Conventional	1/200	>2x SD OD NEC		196	Total of False Negative unclear
	2016	Araujo	Brazil	Conventional	1/300	-	1.1	217	Endemic controls were not included
	2018	Muñoz	Colombia	Conventional	1/200	-	-	438	Total of False Negative unclear
LID-1									
	2010	Duthie	Brazil & Philippines	Conventional	1/999	-		54	It is not a accuracy study
	2011	Duthie	Venezuela	Conventional	1/1000	-	1.1	264	Total of False Negative unclear
	2012	Duthie	Venezuela	Conventional	1/200	>0.2		89	It is not a accuracy study
	2012	Rada	Venezuela	Conventional	1/2500	0.2		205	It is not a accuracy study
	2013	Qiong-Hua	China	Conventional	1/1000	-	-	116	It is not a accuracy study
	2014	Duthie	Colombia & Philippines	Conventional	1/200	>2x SD OD NEC		196	Total of False Negative unclear
	2015	Mizoguti	Brazil	Conventional	1/5000	>2x SD OD EC		50	It is not a accuracy study
	2015	Freitas	Brazil	Conventional	1/200	0.3		30	Endemic controls were not included

OD = Optical Density

EI = ELISA Index

PRISMA 2009 Checklist

Section/topic	#	Checklist item	Reported on page #
**TITLE**			
Title	1	Identify the report as a systematic review, meta-analysis, or both.	Title
**ABSTRACT**			
Structured summary	2	Provide a structured summary including, as applicable: background; objectives; data sources; study eligibility criteria, participants, and interventions; study appraisal and synthesis methods; results; limitations; conclusions and implications of key findings; systematic review registration number.	Abstract
**INTRODUCTION**			
Rationale	3	Describe the rationale for the review in the context of what is already known.	Introduction
Objectives	4	Provide an explicit statement of questions being addressed with reference to participants, interventions, comparisons, outcomes, and study design (PICOS).	Introduction and Methods: Review Question.
**METHODS**			
Protocol and registration	5	Indicate if a review protocol exists, if and where it can be accessed (e.g., Web address), and, if available, provide registration information including registration number.	Methods
Eligibility criteria	6	Specify study characteristics (e.g., PICOS, length of follow-up) and report characteristics (e.g., years considered, language, publication status) used as criteria for eligibility, giving rationale.	Methods: Inclusion Criteria
Information sources	7	Describe all information sources (e.g., databases with dates of coverage, contact with study authors to identify additional studies) in the search and date last searched.	Methods: Search Strategy
Search	8	Present full electronic search strategy for at least one database, including any limits used, such that it could be repeated.	Methods: Search Strategy
Study selection	9	State the process for selecting studies (i.e., screening, eligibility, included in systematic review, and, if applicable, included in the meta-analysis).	Methods: Study Strategy
Data collection process	10	Describe method of data extraction from reports (e.g., piloted forms, independently, in duplicate) and any processes for obtaining and confirming data from investigators.	Methods: Data extraction
Data items	11	List and define all variables for which data were sought (e.g., PICOS, funding sources) and any assumptions and simplifications made.	Methods: Data extraction/Quality assessment
Risk of bias in individual studies	12	Describe methods used for assessing risk of bias of individual studies (including specification of whether this was done at the study or outcome level), and how this information is to be used in any data synthesis.	Methods: Assessment of methodological

PRISMA 2009 Checklist

Summary measures	13	State the principal summary measures (e.g., risk ratio, difference in means).	Methods: Data Synthesis
Synthesis of results	14	Describe the methods of handling data and combining results of studies, if done, including measures of consistency (e.g., I^2) for each meta-analysis.	Methods: Data Synthesis

Section/topic	#	Checklist item	Reported on page #
Risk of bias across studies	15	Specify any assessment of risk of bias that may affect the cumulative evidence (e.g., publication bias, selective reporting within studies).	Methods: Data Synthesis
Additional analyses	16	Describe methods of additional analyses (e.g., sensitivity or subgroup analyses, meta-regression), if done, indicating which were pre-specified.	Methods: Data Synthesis
**RESULTS**			
Study selection	17	Give numbers of studies screened, assessed for eligibility, and included in the review, with reasons for exclusions at each stage, ideally with a flow diagram.	Results (Figure 1)
Study characteristics	18	For each study, present characteristics for which data were extracted (e.g., study size, PICOS, follow-up period) and provide the citations.	Results (Table 1)
Risk of bias within studies	19	Present data on risk of bias of each study and, if available, any outcome level assessment (see item 12).	Results: Figure 2, Table S1
Results of individual studies	20	For all outcomes considered (benefits or harms), present, for each study: (a) simple summary data for each intervention group (b) effect estimates and confidence intervals, ideally with a forest plot.	Results: Figure 3, 4, 5
Synthesis of results	21	Present results of each meta-analysis done, including confidence intervals and measures of consistency.	Results
Risk of bias across studies	22	Present results of any assessment of risk of bias across studies (see Item 15).	Results: Figure S2
Additional analysis	23	Give results of additional analyses, if done (e.g., sensitivity or subgroup analyses, meta-regression [see Item 16]).	Results
**DISCUSSION**			
Summary of evidence	24	Summarize the main findings including the strength of evidence for each main outcome; consider their relevance to key groups (e.g., healthcare providers, users, and policy makers).	Discussion
Limitations	25	Discuss limitations at study and outcome level (e.g., risk of bias), and at review-level (e.g., incomplete retrieval of identified research, reporting bias).	Discussion

Conclusions	26	Provide a general interpretation of the results in the context of other evidence, and implications for future research.	Conclusion
**FUNDING**			
Funding	27	Describe sources of funding for the systematic review and other support (e.g., supply of data); role of funders for the systematic review.	NA

For more information, visit: **www.prisma-statement.org**.

S1 Figure. Methodological quality summary of the included studies

	Risk of Bias				Applicability Concerns		
	Patient Selection	Index Test	Reference Standard	Flow and Timing	Patient Selection	Index Test	Reference Standard
Amorim 2016	+	+	+	+	+	+	+
Britton 1987	+	+	+	+	+	+	+
Cardoso 2013	+	?	+	+	+	+	+
Cellona 1993	+	+	+	+	+	+	+
Chanteau 1991	+	+	+	+	+	+	+
Duthie 2007	+	+	+	+	+	+	+
Frade 2017	+	+	+	+	+	+	+
Freitas 2016	+	+	+	+	+	+	+
Hungria 2012	+	+	+	+	+	+	+
Hungria 2017	+	+	+	+	+	+	+
Moura 2014	+	+	–	+	+	+	+
Praputpittaya 1990	+	+	?	+	+	+	+
Saad 1990	+	+	+	+	+	+	+
Torres 2003	+	+	+	+	+	–	+
Wen 2013	+	+	+	+	+	+	+
Wen 2014	+	+	+	+	+	+	+
Wu 1987	+	+	+	+	+	+	+
Wu 1989	+	?	+	+	+	+	+
Wu 2002	+	+	?	+	+	+	+

– High ? Unclear + Low

S2 Figure. Analysis of publication bias. Deeks' funnel plot for leprosy ELISAs based on different antigens. Deeks' funnel plot (asymmetry test for publication bias) did not detect potential publication bias for A) PGL-I (MB p = 0.63 and PB p = 0.69). B) Only ND-O-BSA in the MB group did not show publication bias (p = 58). C) LID-1 showed publication bias in both groups of patients

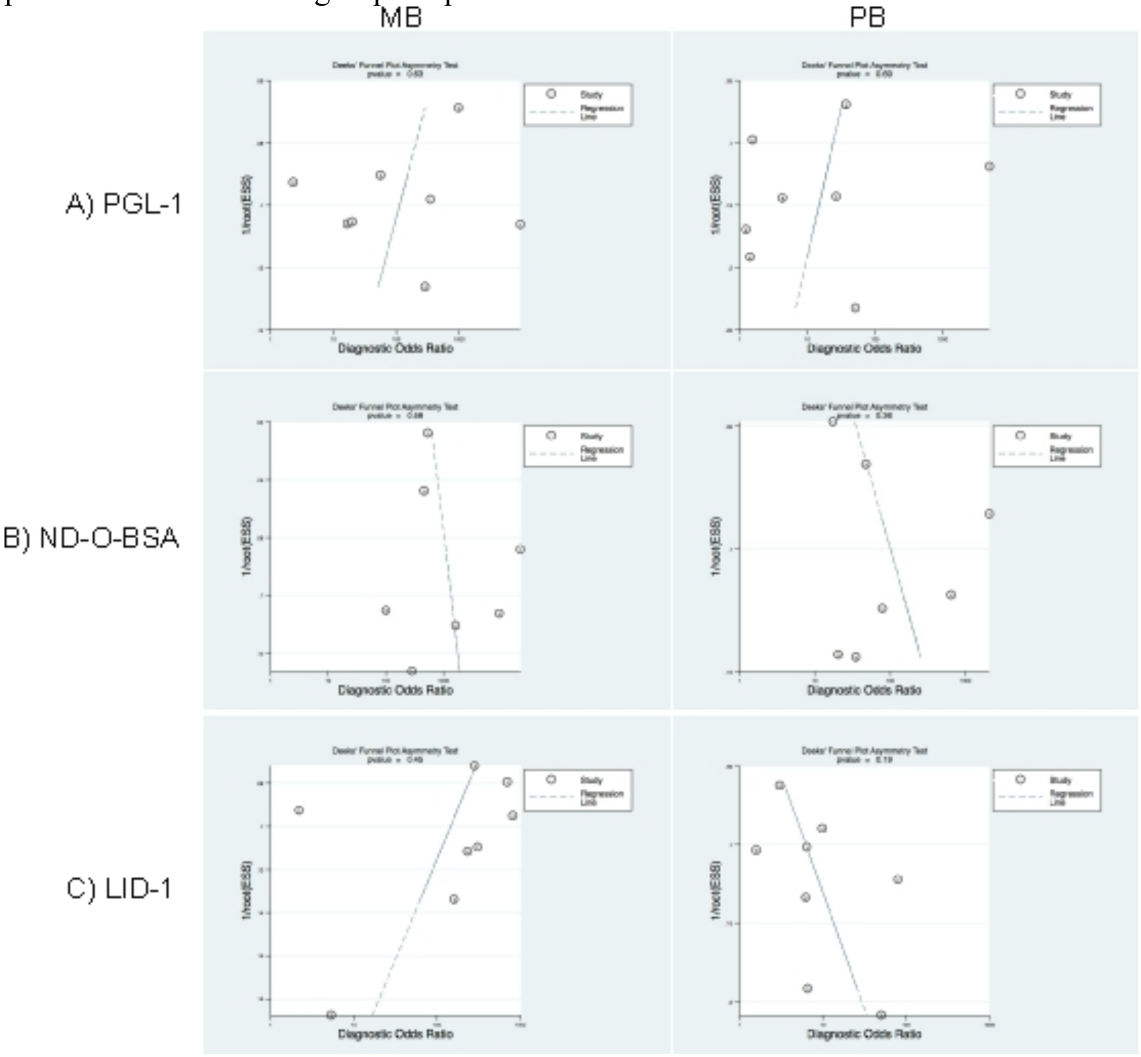

S3 Figure. **Analysis of heterogeneity**. Summary ROC curve plots of sensitivity and specificity for PGL-I (A), ND-O-BSA (B), and LID-1 (C). Each large X represents an individual study in the meta-analysis. The summary operating point is a single sensitivity/specificity point estimated by the results of the studies. AUC = area under the curve.

MB PB

A) PGL-1

B) ND-O-BSA

C) LID-1

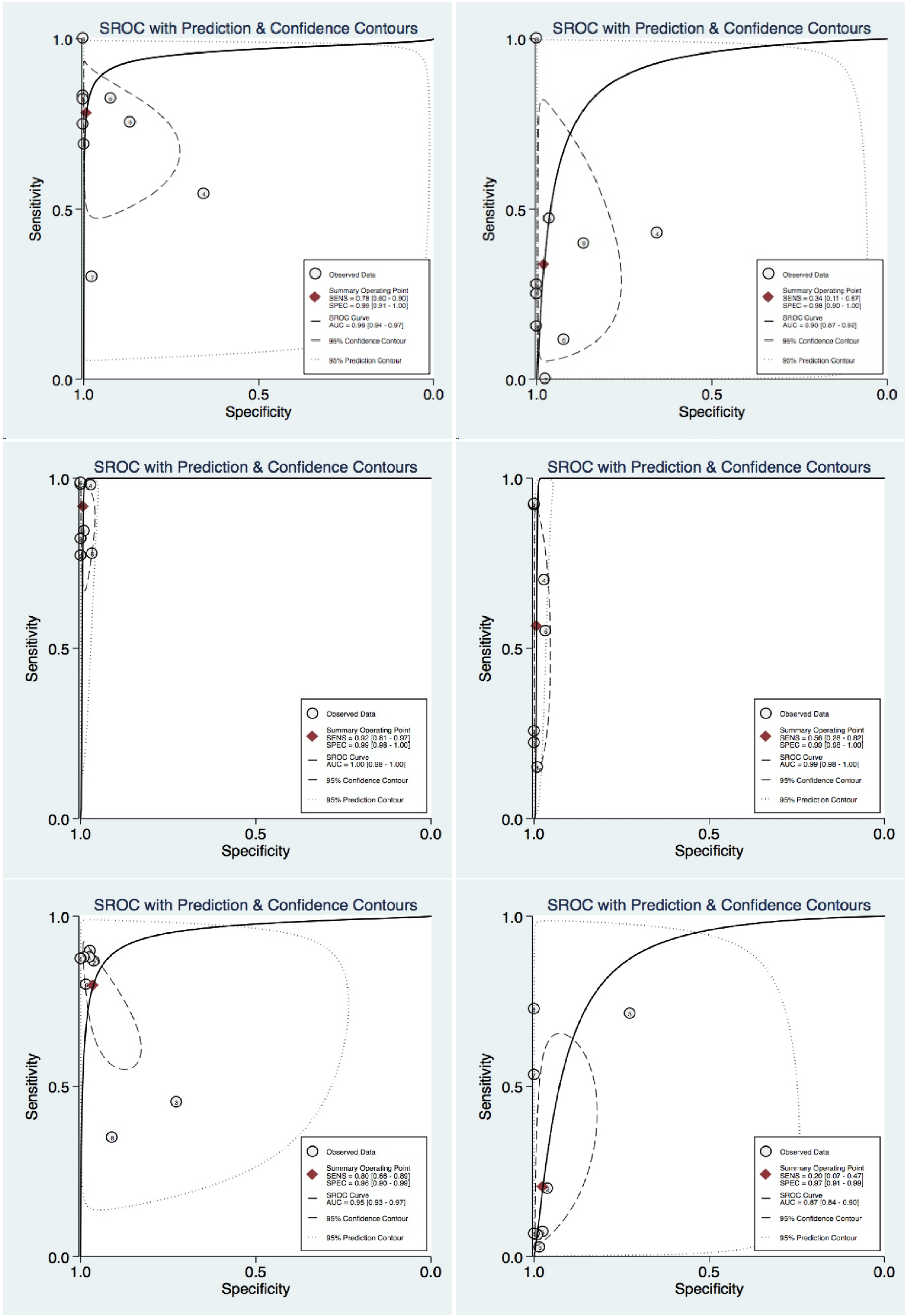

S4 Figure. Meta-analysis based on the hierarchical method. An HSROC plot displaying diagnostic accuracy results of the included studies by antigen in ELISAs: A) PGL-I, B) ND-O-BSA, and C) LID-1 for different leprosy patient groups (MB and PB). The circle diameter (study estimate) is proportional to the weight given to each study. Summary sensitivity and specificity estimates are marked with a red square.

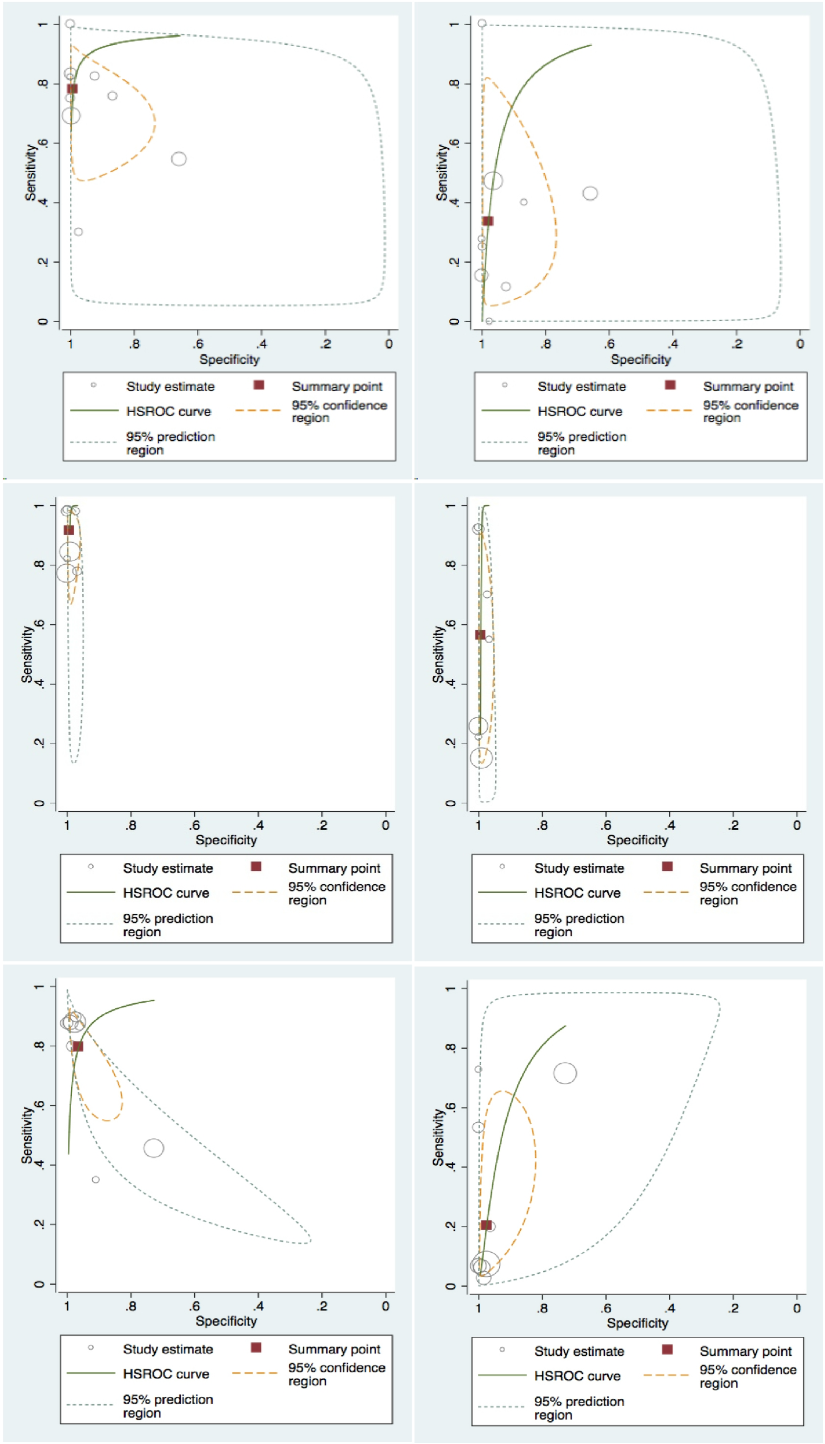

Supplement: Supplementary Materials — Text S1: the protocol registered in Prospero International (PROSPERO 2017: CRD42017055983). Text S2: the literature search strategy. Table S1: a summary of the excluded studies. Table S2: the PRISMA checklist. Figure S1: methodological quality summary of the included studies. Figure S2: analysis of publication bias. Deeks' funnel plot for leprosy ELISAs is based on different antigens. Deeks' funnel plot (asymmetry test for publication bias) did not detect potential publication bias for (A) PGL-I (MB p=0.63 and PB p=0.69). (B) Only ND-O-BSA in the MB group did not show publication bias (p=58). (C) LID-1 showed publication bias in both groups of patients. Figure S3: analysis of heterogeneity. Summary ROC curve plots of sensitivity and specificity for PGL-I (A), ND-O-BSA (B), and LID-1 (C). Each large X represents an individual study in the meta-analysis. The summary operating point is a single sensitivity/specificity point estimated by the results of the studies. AUC = area under the curve. Figure S4: meta-analysis based on the hierarchical method. An HSROC plot displaying diagnostic accuracy results of the included studies by antigen in ELISAs: (A) PGL-I, (B) ND-O-BSA, and (C) LID-1 for different leprosy patient groups (MB and PB). The circle diameter (study estimate) is proportional to the weight given to each study. Summary sensitivity and specificity estimates are marked with a red square. [file 9828023.f1.pdf]
